# Ultramicro Interdigitated Array Electrode Chip with Optimized Construction for Detection of Ammonia Nitrogen in Water

**DOI:** 10.3390/mi14030629

**Published:** 2023-03-10

**Authors:** Haifei Zhao, Yang Li, Aobo Cong, Jianhua Tong, Chao Bian

**Affiliations:** 1 State Key Laboratory of Transducer Technology, Aerospace Information Research Institute, Chinese Academy of Sciences, Beijing 100190, China; zhaohaifei20@mails.ucas.ac.cn (H.Z.); yangli@mail.ie.ac.cn (Y.L.); congaobo20@mails.ucas.ac.cn (A.C.); jhtong@mail.ie.ac.cn (J.T.); 2 School of Electronic, Electrical and Communication Engineering, University of Chinese Academy of Sciences, Beijing 100049, China

**Keywords:** electrochemical sensor, ultramicro interdigitated array electrodes, ammonia nitrogen, generation–collection effect, edge effect

## Abstract

Ammonia nitrogen is a common contaminant in water and its determination is important for environmental protection. In this paper, an electrochemical sensor based on an ultramicro interdigitated array electrode (UIAE) chip with optimized construction was fabricated with Micro-Electro-Mechanical System (MEMS) technology and developed to realize the detection of ammonia nitrogen in water. The effects of spacing-to-width ratio and width of the working electrode on UIAE’s electrochemical characteristics and its ammonia nitrogen detection performance were studied by finite element simulation and experiment. The results demonstrated that the smaller the spacing-to-width ratio, the stronger generation–collection effect, and the smaller the electrode width, the stronger the edge effect, which led to an easier steady-state reach, a higher response current, and better ammonia nitrogen determination performance. The fabricated UIAE chip with optimized construction showed the linear detection range of 0.15 mg/L~2.0 mg/L (calculated as N), the sensitivity of 0.4181 μA·L·mg^−1^, and good anti-interference performance, as well as a long lifetime. UIAE based on bare Pt was successfully applied to ammonia nitrogen detection in water by optimizing structure, which might broaden the methods of ammonia nitrogen detection in water.

## 1. Introduction

Ammonia nitrogen is one of the critical indicators for water quality detection. It refers to nitrogen in the form of free ammonia NH_3_ and ionic ammonium NH_4_^+^. Their proportion in ammonia nitrogen is affected by the pH value and temperature of the solution in real time. The higher the pH value, the higher the temperature, and the greater the proportion of NH_3_ [1]. In addition, ammonia is toxic to humans, fish, and crustaceans, especially aquatic organisms in their growth period and infancy [2,3]. Therefore, the detection of ammonia nitrogen in water is of great significance to safeguard human health and protect the ecosystem.

Traditional methods for ammonia nitrogen detection mainly include spectrophotometry and fluorescence, but there are several problems such as cumbersome test steps, long test cycles, the use of many reagents, and susceptibility to interference [4,5,6]. Electrochemical methods have advantages in field rapid detection due to their ease of use, rapid response, and strong anti-interference capabilities. In electrochemical methods, the construction of highly sensitive catalysts is crucial. For ammonia, the sensitive materials can be categorized as conductive polymers, carbon-based materials, nanometal, nanometal oxides, and the combinations of them [7,8,9,10,11,12,13,14,15,16,17,18,19]. Pt has been considered to be the metal catalyst with the best performance for ammonia electro-oxidation [20,21]. However, during the electro-oxidation of ammonia, the intermediate products are easily adsorbed on the electrode surface, which can lead to the poisoning of the electrode [22,23]. To address this issue, Pt-based nanoparticles and nanocomposites were developed, such as Pt NPs-Pt electrode [12,13], Pt NPs-ITO electrode [14], PtNi NPs-CC electrode [15], PtCu NPs-CC electrode [16], PtAg NPs-PPy-NiF composite electrode [17], Pt NPs-Ni(OH)_2_-CC composite electrode [18], Pt NPs-PANI-CC composite electrode [19], etc. These nanomaterials can provide more active sites for the oxidation of ammonia to reduce poisoning and passivation of the electrodes. However, the shortcomings of the modified nanomaterials are also apparent. Modified nanomaterials are commonly prone to exfoliation, which can affect the stability and lifetime of the electrodes. How to ensure the lifetime of the sensor while maintaining its ability to sensitively detect ammonia nitrogen is crucial.

Interdigitated array electrodes have been extensively studied in the field of biochemical detection because of their high mass transfer rate, high signal-to-noise ratio, high response current, and high sensitivity [24,25]. The spacing of adjacent working electrodes affects the shielding effect and the generation–collection effect of micro interdigitated array electrodes. The shielding effect refers to the phenomenon of current reduction due to the overlapping of the diffusion bands of each working electrode with each other, which is most obvious in the microband array electrodes. The overlapping occurs if the thickness of the diffusion layer is greater than half of the working electrode spacing during the electrochemical reaction [26,27]. For reversible redox reactions, there is a generation–collection effect occurring at the interdigitated array electrodes. That is, the oxidation state and reduction state of the reactant proceed the redox cycling between the two groups of electrode arrays with the change in potential. The generation–collection effect can improve the mass transfer rate and response current [28]. The smaller the spacing, the stronger the redox cycling. In addition, the width of the working electrode is a key factor in determining the strength of the edge effect. A smaller width results in stronger edge effect, with nonlinear diffusion becoming the dominant mechanism, leading to a higher response current [29].

In this study, ammonia nitrogen was detected using the UIAE chip fabricated with MEMS technology. Unmodified bare Pt, which can achieve simple operation, good stability, and a long lifetime, was used as the sensitive material. In addition, the spacing-to-width ratio and the width of the working electrodes were optimized to minimize electrode poisoning and improve the electro-oxidation performance of ammonia.

## 2. Simulation and Experiment

### 2.1. Instruments and Reagents

A Gamry Reference 600 electrochemical workstation (Gamry, Warminster, PA, USA), electronic balance (Sartorius, Göttingen, Germany), ultrapure water machine (Beijing Yingan Meicheng Scientific Instrument Co., Ltd., Beijing, China), and Ag/AgCl reference electrode (Shanghai Chenhua Instrument Co., Ltd., Shanghai, China) were employed. Finite element analysis software was used to simulate the electrochemical performance of the ultramicro array electrode chip.

Potassium chloride (KCl), potassium ferricyanide (K_3_[Fe(CN)_6_]), potassium ferrocyanide trihydrate (K_4_[Fe(CN)_6_]), sodium hydroxide (NaOH), disodium hydrogen phosphate (Na_2_HPO_4_), and ammonium sulfate ([NH_4_]_2_SO_4_) were purchased from Sinopharm Chemical Reagent Co., Ltd. (Shanghai, China). All experimental reagents were analytically pure, and the experimental water was deionized water made from ultrapure water. Unless otherwise specified, the experimental temperature conditions were room temperature (25 °C).

### 2.2. Ultramicro Array Electrode Chip Design and Simulation

**Figure 1:**
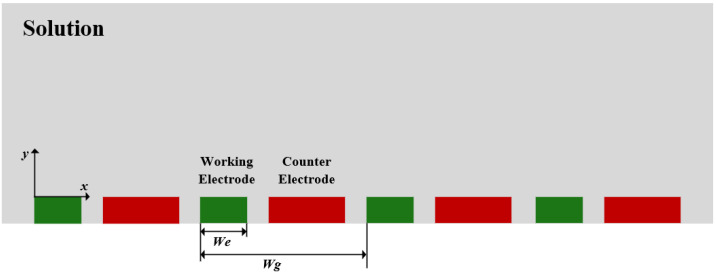
Modeling schematic diagram of UIAE (*We*, the width of the working electrode; *W_g_*, the spacing between the working electrodes).

The length of the array electrodes was set to 2 mm, which was much larger than the width and height of the electrodes with a micrometer level, so the two-dimensional spatial dimension was used for modeling. The height of the Pt UIAE was set to 200 nm. The width of the counter electrode was set to twice that of the working electrode to ensure that the counter electrode had a larger area than the working electrode to prevent polarization. The modeling schematic diagram was shown in Figure 1.

In the simulation, the one-electron reversible reaction in solution was considered as Equation (1).

(1) $${O+e}^{-}↔\;R$$

It was assumed that the electron transfer rate of the electrode was controlled by Butler–Volmer kinetics as in Equation (2).

(2) $$D_{O}\frac{\partial C_{O}}{\partial x}=-D_{R}\frac{\partial C_{R}}{\partial y}=k_{0}\left(C_{R}exp\left(-\alpha \theta \right)-C_{O}exp\left(\left(1-\alpha \right)\theta \right)\right)$$

where *k*_0_ is a non-uniform rate constant; α is the charge transfer coefficient; *D* is the diffusion coefficient of the substance; and *D_O_* and *D_R_* represent the diffusion coefficients of substances *O* and *R*, respectively. This paper assumed that *D_O_* = *D_R_* = 7.6 × 10^−6^ cm^2^/s.

The mass transfer equation was:

(3) $$\frac{\partial C}{\partial t}=D\left(\frac{\partial^{2}C}{\partial x^{2}}+\frac{\partial^{2}C}{\partial y^{2}}\right)$$

The boundary conditions were:

(4) $$\mathrm{When}\;t\;=\;0,\;x\;\mathrm{and}\;y\;\mathrm{were}\;\mathrm{arbitrary}\;\mathrm{values},\;C_{O}=C_{O}^{*},\;C_{R}=C_{R}^{*}$$

(5) $$\mathrm{When}\;t\;>\;0,\;y\mathrm{was}\;\mathrm{arbitrary}\;\mathrm{values},\;\underset{x\to \infty }{lim}C_{O}=C_{O}^{*},\;\underset{x\to \infty }{lim}C_{R}=C_{R}^{*}$$

(6) $$\mathrm{When}\;t\;>\;0,\;x\mathrm{was}\;\mathrm{arbitrary}\;\mathrm{values},\;\underset{y\to \infty }{lim}C_{O}=C_{O}^{*},\;\underset{y\to \infty }{lim}C_{R}=C_{R}^{*}$$

where $C_{O}$ and $C_{R}$ are the concentrations of substance *O* and substance *R* at any time and any position, respectively. $C_{O}^{*}$ and $C_{R}^{*}$ are the bulk solution concentrations of substance *O* and substance *R*, respectively. Initial values of $C_{O}^{*}$ and $C_{R}^{*}$ were set 0 mM and 0.5 mM, respectively.

The current density of this reaction was given by the Butler–Volmer equation:

(7) $$i_{loc\;}=nFk_{0}\left(C_{R}exp\left(\frac{\left(n-\alpha_{c}\right)F\eta }{RT}\right)-C_{O}exp\left(\frac{-\alpha_{c}F\eta }{RT}\right)\right)$$

where α*_c_* is the cathode transfer coefficient, *η* is the overpotential of the working electrode, and *n* is the number of electrons transferred.

**Figure 2:**
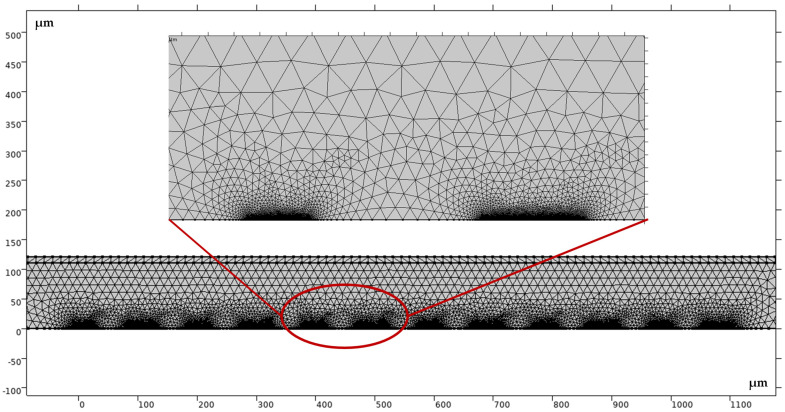
Finite element mesh generation diagram.

In this paper, the electrode domain of interdigital array electrode was finely divided, while other domains were relatively coarsely divided, and the results were shown in the Figure 2.

When simulating the influence of the spacing-to-width ratio on the electrochemical reaction, the parametric scanning of the spacing-to-width ratio was set to 5~15 with a step size of 2, and the working electrode width was fixed at 15 μm. To shorten the solution time, the number of arrays was set to 6. When simulating the effect of electrode width on the electrochemical reaction, the widths were set to 5 μm, 10 μm, and 15 μm, respectively, and the number of arrays was set to 18, 9, and 6, which ensured the consistent working-electrode area, and the spacing-to-width ratio was fixed at 13.

For comparison, the electrochemical reactions of both UIAE and ultramicro band array electrodes (UBAE) were simulated, respectively. When simulating the character of UIAE, the counter-electrode array was enabled. The boundary condition of the working-electrode surface was set to cyclic voltammetry with a starting vertex potential of −0.2 V, an ending vertex potential of 0.6 V and a scanning rate of 50 mV/s. To simulate the generation–collection effect, the boundary conditions of the counter-electrode surface were also set to cyclic voltammetry with a starting vertex potential of 0.6 V, an ending vertex potential of −0.2 V, and a scanning rate of 50 mV/s. When simulating the character of UBAE, the counter-electrode array of UIAE was disabled, and the boundary condition of the working-electrode surface was set as that of the UIAE.

### 2.3. Ultramicro Interdigitated Array Electrode Chip Fabrication

UIAE chips with different spacing-to-width ratios and different working-electrode widths were fabricated with MEMS technology. The specific process was as follows: Firstly, 800 nm of silicon oxide and 200 nm of silicon nitride were deposited on a 4-inch silicon wafer as a hybrid insulating layer with Low-Pressure Chemical Vapor Deposition (LPCVD). Secondly, the positive photoresist AZ1500 was coated on the silicon wafer, and formed the pattern of the electrode by photolithography. Next, titanium (Ti) with a thickness of 20 nm was sputtered as the adhesion layer, and then platinum (Pt) with a thickness of 200 nm was sputtered as the electrode layer, and the pattern transfer was completed by the lift-off process. After that, a second photolithography was performed to cover the negative photoresist SU-8 as an insulating layer. Finally, after dicing, pressure welding, and packaging, the UIAE chips were fabricated and ready for use.

**Figure 3:**
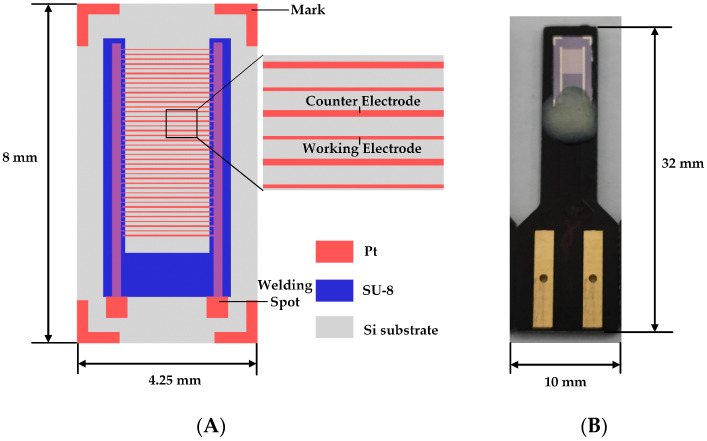
UIAE chip: (**A**) Structure diagram; (**B**) Photograph.

The structural diagram and photograph of the fabricated UIAE chip are shown in Figure 3A,B, respectively. The sizes of the UIAE chip before and after packaging are 8 mm × 4.25 mm and 32 mm × 10 mm, respectively. About 160 small chips can be obtained from one 4-inch silicon wafer. Therefore, it has the advantage of cost compared with the commercially available sensing devices. The working electrode and the counter electrode were in an interdigitated array structure. The length, width, and spacing-to-width ratio of the electrode were in accordance with the simulation, except the number of electrode arrays. The number of electrode arrays during the experiment was increased compared with the simulation process to obtain a high-current response. Moreover, to ensure a consistent effective working area, different numbers of arrays were designed according to different electrode widths. The effective area of the working electrode with different configuration was 0.6 mm^2^ invariably. Before each electrochemical test, the electrode chip was placed in 0.1 M H_2_SO_4_, and cyclic voltammetric scanning at −0.6 V~1.0 V was performed to remove impurities and oxide layers from the electrode surface and activate the electrode.

### 2.4. Electrochemical Characterization and Ammonia Nitrogen Determination

Cyclic voltammetry (CV) was used to characterize the electrochemical performance of the ultramicro array electrode chip in a 0.5 mM Fe(CN)_6_^3−^/Fe(CN)_6_^4−^ and 0.1 M KCl solution with the scanning range of −0.2 V~0.6 V.

(NH_4_)_2_SO_4_ was used to prepare an ammonia nitrogen solution. The base solution with pH 11 (converting ionic ammonium into free ammonia) was prepared by adding 2 g NaOH to 1 L of 0.2 M Na_2_HPO_4_. Ammonia nitrogen solutions with the concentrations of 0.15 mg/L, 0.5 mg/L, 1.0 mg/L, 1.5 mg/L, and 2.0 mg/L (calculated as N) were prepared to simulate five categories of surface water according to the Environmental Quality Standard for Surface Water GB 3838-2002 of China. The UIAE chip and an external Ag/AgCl reference electrode were used to construct the electrochemical sensor for ammonia nitrogen detection. The Differential Pulse Voltammetry (DPV) method was used with the voltage range of −0.5 V~0.1 V, the pulse amplitude of 50 mV, the pulse width of 0.02 s, and the pulse period of 0.05 s.

## 3. Results and Discussion

### 3.1. Simulation Results and Analysis of Ultramicro Array Electrodes

#### 3.1.1. Simulation Comparison between UIAE and UBAE

**Figure 4:**
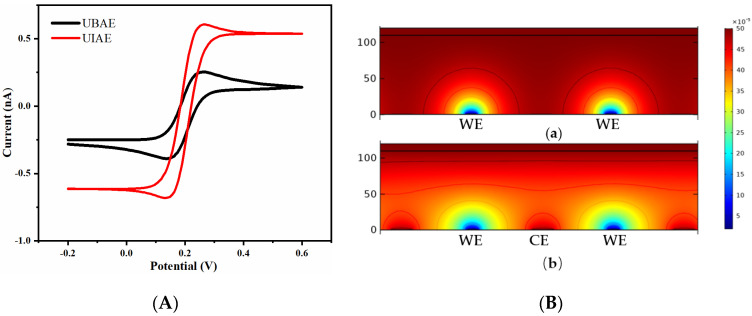
Simulation comparison results of UBAE and UIAE with spacing-to-width ratio of 13: (**A**) cyclic voltammogram; (**B**) concentration distribution diagram: (**a**), UBAE; (**b**), UIAE.

The simulation comparison results of UIAE and UBAE with the same spacing-to-width ratio of 13 and the same working-electrode width of 15 μm are shown in Figure 4. The CV characterization and concentration distribution diagram of UIAE and UBAE were simulated. As shown in Figure 4A, under this condition, the CV curves of UIAE and UBAE are close to a sigmoidal shape. Compared with UBAE, the current response of UIAE reaches steady-state more easily and its response value is higher.

As shown in the concentration distribution diagram of UBAE (Figure 4B(a)), there is no overlap between the diffusion layers of the adjacent working electrode, which displays a radial diffusion without the affection of the shielding effect. Form the concentration profile of UBAE, the sigmoidal-shape CV response of UBAE can be explained.

As shown in the concentration distribution diagram of UIAE (Figure 4B(b)), the diffusion layer between the working and counter electrode is in a state of overlap due to the reversible reaction on the surface of the interdigitated electrode, which can promote the generation–collection effect. Under the Fe(CN)_6_^3−^/Fe(CN)_6_^4−^ system, this can be explained as follows: the overlapping of the diffusion layers promotes the rapid diffusion of Fe(CN)_6_^3−^/Fe(CN)_6_^4−^ between the working electrode and counter electrode of UIAE and provides sufficient electroactive solution ions; therefore, the working electrode can supply the counter electrode with extra Fe(CN)_6_^3−^, whereas the counter electrode can supply the working electrode with extra Fe(CN)_6_^4−^ (anodic sweep), resulting in steady-state rather than an obvious decrease in current [30]. Therefore, UIAE reaches steady-state more easily and has a higher current response than UBAE.

#### 3.1.2. Simulation Results and Analysis of UIAE with Different Spacing-to-Width Ratios

**Figure 5:**
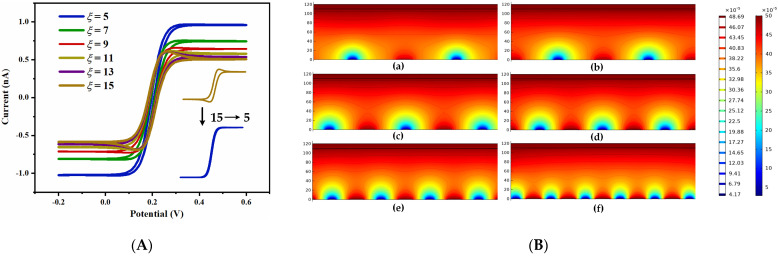
Simulation results diagrams of UIAE with different spacing-to-width ratios: (**A**) cyclic voltammogram; (**B**) concentration distribution diagram: (**a**), 15; (**b**), 13; (**c**), 11; (**d**), 9; (**e**), 7; (**f**), 5.

The CV and concentration distribution diagram of UIAE with different spacing-to-width ratios were simulated. As shown in Figure 5A, as the spacing-to-width ratio decreases from 15 to 5, the oxidation peak current gradually increases, and the closer the shape of the CV curve is to the typical sigmoidal shape of the ultramicro electrode and the easier it is to reach steady-state. According to the results of Figure 5B, with the decrease in the spacing-to-width ratio, the diffusion overlapping between the working and counter electrode becomes more obvious, and the concentration of substances generated on the counter electrode increases continuously, indicating that the smaller the spacing-to-width ratio, the more easily the substances generated on the electrode surface can diffuse to the adjacent electrodes to react. This can be explained in that the smaller the spacing-to-width ratio of UIAE, the stronger the generation–collection effect is, the faster the electrochemical substance diffuses between adjacent electrodes, and there are enough solution ions to participate in the electron transfer process of the electrodes, thus improving the response current and achieving steady-state diffusion more easily. Inversely, the larger the spacing-to-width ratio, the lower the current response. When the spacing-to-width ratio is larger than 11, the peak current value basically remains stable, which means that the larger the spacing-to-width ratio, the weaker the generation–collection effect is until it fails.

#### 3.1.3. Simulation Results and Analysis of UIAE with Different Widths of Electrode

**Figure 6:**
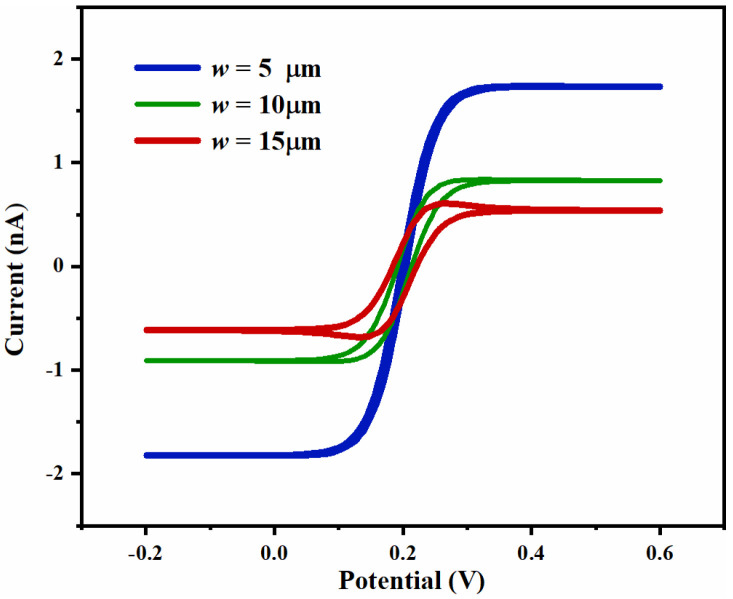
Simulated CV curves of UIAE with different electrode widths.

The simulated CV curves of the UIAE with working-electrode widths of 5 μm, 10 μm, and 15 μm are shown in Figure 6. The smaller the width of the working electrode, the larger the peak current, and the CV curve gradually tends towards the typical sigmoidal shape. It can be explained that stronger edge effect guides the nonlinear diffusion on the electrode surface, leading to a high mass transfer rate and adequate ions, further reaching steady-state easily.

### 3.2. Electrochemical Characterization of Array Electrodes

#### 3.2.1. Comparison between UIAE and UBAE

The electrochemical CV characteristics of UBAE and UIAE were investigated and compared. The fabricated UIAE chip with the spacing-to-width ratio of 13 was used. UBAE was detected by using an on-chip working-electrode array and external columnar Pt counter electrode.

**Figure 7:**
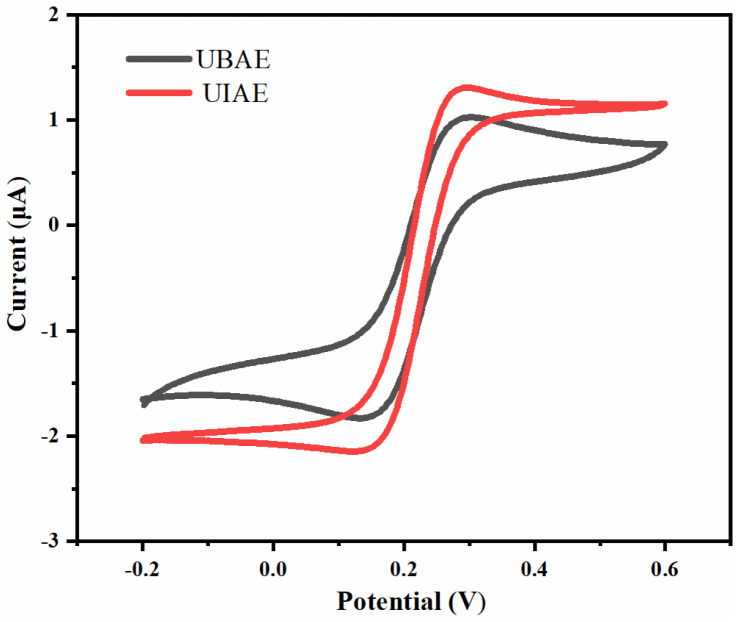
Comparison of electrochemical cyclic voltammetric characteristics between UIAE and UBAE with the spacing-to-width ratio of 13.

As shown in Figure 7, the CV curve of UIAE displays a closer sigmoidal shape and a higher peak current than UBAE. Combined with the simulation results, this can be attributed to the generation–collection effect of UIAE, which improves the mass transfer rate and replenishes reactive ions.

#### 3.2.2. Effect of Spacing-to-Width Ratio of UIAE on Cyclic Voltammetry Characteristics

**Figure 8:**
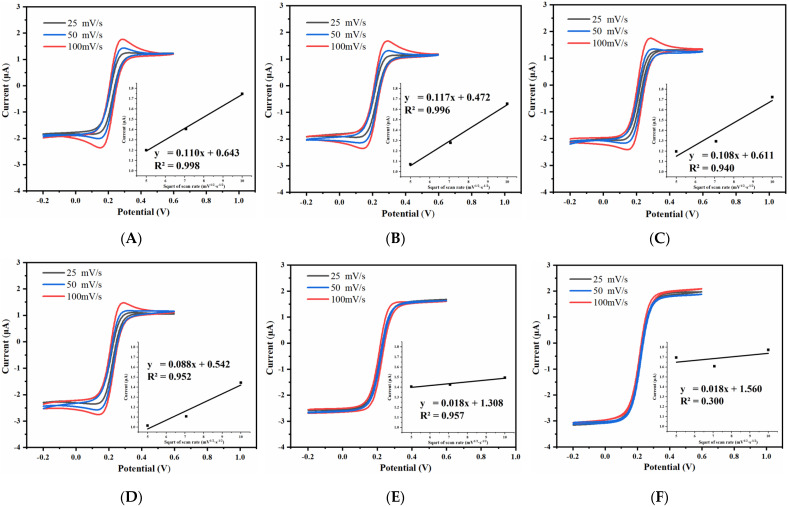
Cyclic voltammogram and the relationship between the oxidation peak current and the square root of scanning rate (inset) of UIAE with different spacing-to-width ratios: (**A**) 15; (**B**) 13; (**C**) 11; (**D**) 9; (**E**) 7; (**F**) 5.

The cyclic voltammetric response of the prepared UIAE with different spacing-to-width ratio was tested, and the effect of scanning rate on the cyclic voltammetric peak current of the electrode was studied. The scanning rate was set at 25 mV/s, 50 mV/s, and 100 mV/s, respectively. The results are shown in Figure 8.

As shown in Figure 8, the cyclic voltammetric peak current of the electrodes with the spacing-to-width ratios of 5 and 7 show no obvious change with the change in scanning rate. In addition, their cyclic voltammetric curves are the typical sigmoidal shape of ultramicro electrodes. The peak current value of each UIAE clearly changes at different scanning rates as the spacing-to-width ratio of the UIAE increases, which gradually shows a good linearity between the peak current value and the square root of the scanning rate, indicating that the reaction is gradually controlled by diffusion steps and the electrode surface reaction is gradually dominated by linear diffusion.

**Figure 9:**
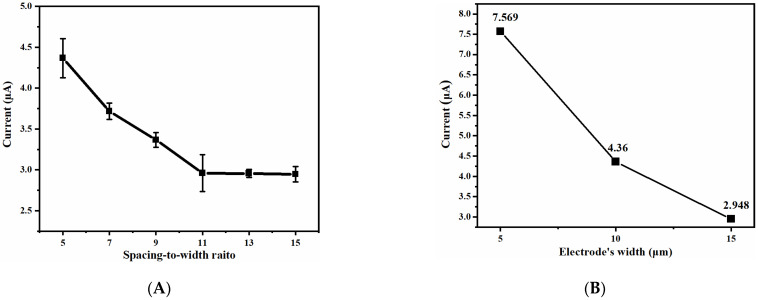
Relationship between response current and (**A**) spacing-to-width ratio; (**B**) width of UIAE.

Taking the difference between the oxidation peak current and the reduction peak current as a measure of the response current, when the scanning rate is 50 mV/s, the relationship between the response current and the spacing-to-width ratio is shown in Figure 9A. The UIAE with the small spacing-to-width ratio has large response current, and the current value decreases as the spacing-to-width ratio increases. Therefore, the smaller the spacing-to-width ratio is, the more dominant the generation–collection effect is compared with the shielding effect. When the spacing-to-width ratio increases to 11, the response current basically remains the same, which is also consistent with the simulation results. It can be considered that the smaller the spacing-to-width ratio of UIAE, the more rapid the diffusion of Fe(CN)_6_^3−^/Fe(CN)_6_^4−^ between working electrode and counter electrode of UIAE is, producing extra Fe(CN)_6_^3−^/Fe(CN)_6_^4−^, further leading to the higher response current. If the spacing-to-width ratio is large enough, as in the case of more than 11 in this experiment, the generation–collection effect and shielding effect may fail, leaving the response current nearly unchanged.

#### 3.2.3. Effect of Width of UIAE on Cyclic Voltammetry Characteristics

**Figure 10:**
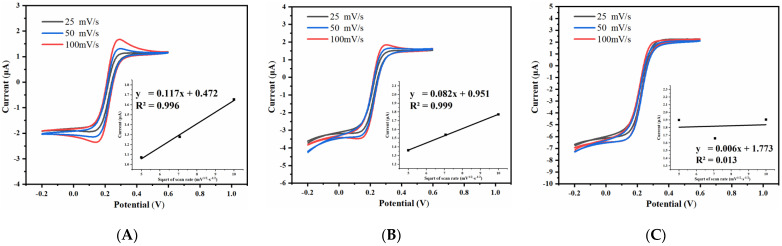
Cyclic voltammogram and the relationship between the oxidation peak current and the square root of scanning (inset) of UIAE with different widths: (**A**) 15 μm; (**B**) 10 μm; (**C**) 5 μm.

The effect of electrode width on the cyclic voltammetric characteristic was studied under the condition of maintaining the same spacing-to-width ratio of 13. The cyclic voltammograms of the UIAE with working-electrode widths of 15 μm, 10 μm, and 5 μm at various scanning rates are shown in Figure 10.

As shown in Figure 10, with the decrease in the working-electrode width from 15 μm to 5 μm, the CV oxidation peak current shows no obvious change with the scanning rate. In addition, the CV curve with the working-electrode width of 5 μm is closer to the typical sigmoidal shape of the ultramicro electrodes, indicating that nonlinear diffusion is gradually taking over the electrode surface. At a scanning rate of 50 mV/s, the correlation between the current difference between the oxidation and reduction peaks and the electrode width is shown in Figure 9B. The narrower the working electrode, the greater the response current. This can be attributed to the fact that the smaller the working-electrode width, the stronger the edge effect, which causes the electrode surface to be dominated by nonlinear diffusion and thus obtain a high mass transfer rate. A higher mass transfer rate allows for more ions to participate in the reaction, resulting in a higher response current.

The above experimental results are consistent with the simulation results, indicating that UIAE with a small spacing-to-width ratio and small width are favorable to exhibit the ultramicroelectrode characteristics dominated by nonlinear diffusion and a better generation–collection effect.

### 3.3. Detection of Ammonia Nitrogen with UIAE

#### 3.3.1. Effect of Spacing-to-Width Ratio on Ammonia Nitrogen Detection Performance

**Figure 11:**
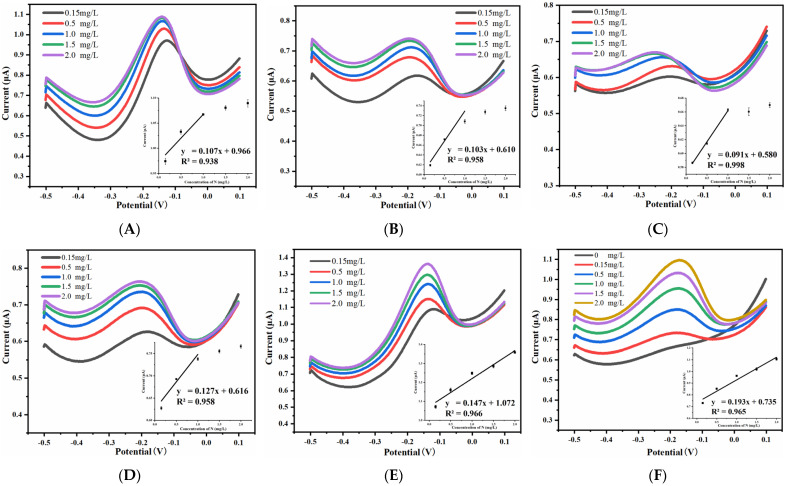
DPV response curves and the calibration curves (inset) of UIAE chips for NH_3_-N with different spacing-to-width ratios: (**A**) 15; (**B**) 13; (**C**) 11; (**D**) 9; (**E**) 7; (**F**) 5.

The UIAE chips with different spacing-to-width ratios were used for the electrochemical detection of ammonia nitrogen. DPV response curves and current–concentration fitting lines are shown in Figure 11. It was found that UIAE chips with spacing-to-width ratios of 5 and 7 demonstrate better performance on detecting ammonia nitrogen in five different concentrations. The UIAE chip with spacing-to-width ratio larger than 7 is not sensitive to high concentrations, and the current is slightly higher or nearly coincident with the response current of 1.0 mg/L ammonia nitrogen. Therefore, only the low concentration range (0.15 mg/L, 0.5 mg/L, and 1.0 mg/L) is linearly fitted. In order to observe the regular pattern more visually, the detection performance of the UIAE with different spacing-to-width ratios are summarized in Table 1.

In addition, it can be seen that an obvious peak which is attributed to the oxidation of ammonia to nitrogen is observed based on Pt UIAE with different spacing-to-width ratios from Figure 11. The complex process of the ammonia electro-oxidation reaction was discussed via Equations (8)–(13) [21,22,23]. Firstly, NH_3_ in the solution (NH_3, aq_) absorbs on the surface of working electrodes to form NH_3, ads_. Then, various intermediates of nitrogen species with successive dehydrogenation form N_2_. As shown in Table 1, when the working-electrode width is 15 μm, with the increased spacing-to-width ratio of the UIAE, the detection range for ammonia nitrogen tends to be narrow, and the sensitivity gradually decreases. It can be considered that the generation–collection effect of UIAE is beneficial to the catalytic oxidation of ammonia. The generation–collection effect here can be explained as follows: during the anodic sweep, the low-valent N compounds are continuously dehydrogenated by the oxidation reaction on the working electrode’s surface, and the products diffuse to the counter electrode to be again reduced to the low-valent N compounds, which then diffuse to the working electrode to continue the oxidation reaction. Therefore, NH_3_ and intermediate products can be more efficiently converted into the final product N_2_ through continuous oxidation.

NH_3, aq_ → NH_3, ads_ (8)

NH_3, ads_ + OH^−^ → NH_2, ads_ + H_2_O + e^−^ (9)

NH_2, ads_ + OH^−^ → NH_ads_ + H_2_O + e^−^ (10)

NH_ads_ + OH^−^ → N_ads_ + H_2_O + e^−^ (11)

NH_x, ads_ + NH_y, ads_ → H_x_NNH_y, ads_ (12)

H_x_NNH_y, ads_ + (x + y)OH^−^ → N_2_ + (x + y)H_2_O + (x + y)e^−^ (13)

where x = 0, 1 or 2; y = 0, 1 or 2.

The smaller the spacing-to-width ratio, the stronger the catalytic effect on ammonia. On the contrary, UIAE with a large spacing-to-width ratio may be more likely to generate intermediate adsorbate in the process of catalyzing ammonia because of the poor generation–collection effect, which makes the electrode exhibit insensitivity to ammonia nitrogen samples, especially to high-concentration samples. In conclusion, the UIAE with a small spacing-to-width ratio is more efficient in detecting ammonia nitrogen.

#### 3.3.2. Effect of Width on Ammonia Nitrogen Detection Performance

**Figure 12:**
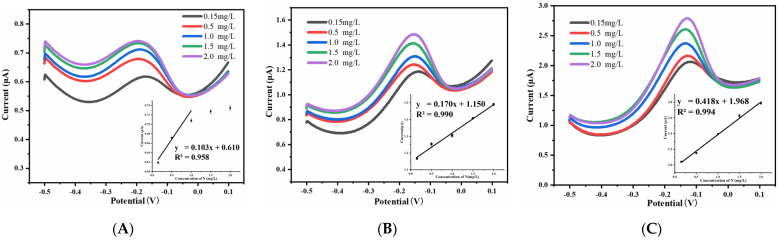
DPV response curves and the calibration curves (inset) of UIAE chips for NH_3_-N with different widths: (**A**) 15 μm; (**B**) 10 μm; (**C**) 5 μm.

Retaining the consistent spacing-to-width ratio, the UIAE chips with different widths of working electrode were used for the electrochemical detection of ammonia nitrogen. With the spacing-to-width ratio of 13, DPV response curves and current–concentration fitting lines are shown in Figure 12. It can also be seen that an obvious peak occurs at the DPV curve when the width is 5 μm or 10 μm, which indicates that these UIAE can also detect ammonia nitrogen. For easy comparison, the detection results are also presented in Table 1.

As shown in Table 1, the smaller the working-electrode width, the wider the linear range, the higher the sensitivity, and the better the linearity of the UIAE chip for ammonia nitrogen detection. This can be explained by the fact that the smaller the working-electrode width, the better the catalytic performance for free ammonia due to its stronger edge effect, thus reducing the poisoning of the electrode.

#### 3.3.3. Other Performance Tests

The electrochemical sensor based on the UIAE chip with the working-electrode width of 5 μm, the spacing-to-width ratio of 13, and the array number of 60 was chosen for the anti-interference test. Its repeatability and stability were evaluated as well. 

**Figure 13:**
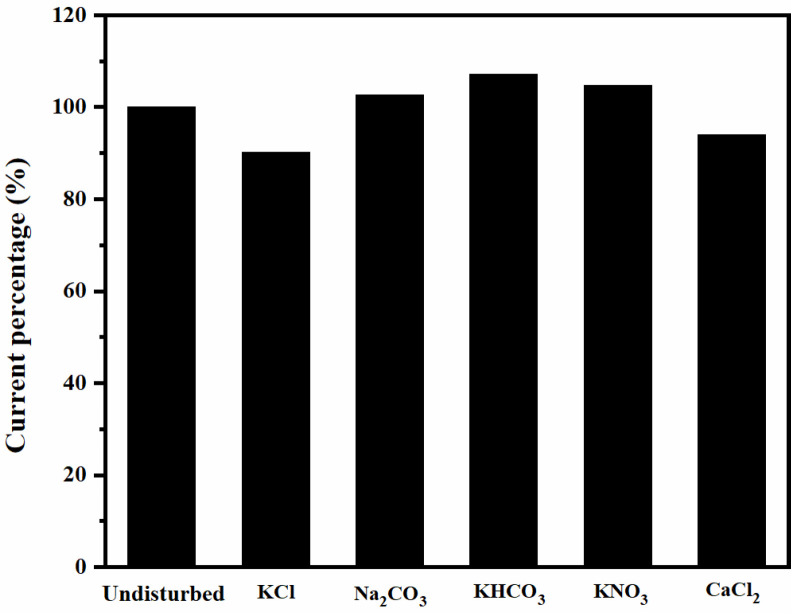
Influence of different interfering ions on response current.

As shown in Table 1 and Figure 12, this sensor chip showed typical ultramicro electrode characteristics, good linear detection range, and sensitivity for NH_3_-N detection. The anti-interference capacity of this sensor chip to water-commonly occurring Cl^−^, K^+^, CO_3_^2−^, HCO_3_^−^, NO_3_^−^, and Ca^2+^ (deriving from KCl, Na_2_CO_3_, KHCO_3_, KNO_3_, and CaCl_2_) was investigated. In a 1 mg/L (calculated as N) ammonia nitrogen solution, these interfering ions with concentrations 10 times higher than ammonia nitrogen (0.714 mmol/L) were added separately and the corresponding current response of the sensor electrode chip was measured. The response currents were compared with those of the 1 mg/L ammonia nitrogen solution without interfering ions, and the testing results are shown in Figure 13.

The deviations of current response caused by adding these interference ions are within 10%, which indicates that the sensor chip has an acceptable anti-interference ability with respect to Cl^−^, K^+^, CO_3_^2−^, HCO_3_^−^, NO_3_^−^, and Ca^2+^.

The NH_3_-N solution of 1mg/L was measured 20 times, and the relative standard deviation (RSD) of the peak currents was 2.90%. In addition, it had been about six months since the initial use of the chip to the latest test, and the change in sensitivity for ammonia nitrogen detection was 7.66%. These results show that the sensor also displays excellent repeatability, great stability, and a long lifetime for ammonia nitrogen detection.

## 4. Conclusions

In summary, the effects of two UIAE characteristic parameters: spacing-to-width ratio and working-electrode width, on the response in the Fe(CN)_6_^3−^/Fe(CN)_6_^4−^ reversible system and in ammonia nitrogen solution were studied by finite element simulation and experiment. The results show that the UIAE chip with smaller spacing-to-width ratio and smaller working-electrode width can reach steady-state more easily and obtain a higher response current. The UIAE chip with optimized structure has an excellent linear response and anti-interference ability for ammonia nitrogen in water. In addition, bare Pt-based UIAE prepared with MEMS technology can obtain low cost, simple operation, and a long lifetime, which also shows the good potential for its application to detect ammonia nitrogen in water. Finally, this paper also provides some guidance for the interdigitated array electrode structure design, which can be used to sensitively detect other ions by changing electrode materials or selecting the appropriate voltammetric analysis methods.

## Figures and Tables

**Table 1:** Results of NH_3_-N detection using UIAE with various spacing-to-width ratios and widths.

Width (μm)	Spacing-To-Width Ratio	Detection Range (mg/L)	Sensitivity (μA·L·mg^−1^)	Linearity
15	15	0.15~1.0	0.107	0.938
	13	0.15~1.0	0.103	0.958
	11	0.15~1.0	0.091	0.998
	9	0.15~1.0	0.127	0.958
	7	0.15~2.0	0.147	0.966
	5	0.15~2.0	0.193	0.965
10	13	0.15~2.0	0.170	0.990
5	13	0.15~2.0	0.418	0.994

## Data Availability

The data that support the findings of this study are available from the corresponding author upon reasonable request.
